# Efficacy and safety of single-dose ivermectin in mild-to-moderate COVID-19: the double-blind, randomized, placebo-controlled CORVETTE-01 trial

**DOI:** 10.3389/fmed.2023.1139046

**Published:** 2023-05-22

**Authors:** Tatsuhiko Wada, Makoto Hibino, Hiromi Aono, Shunsuke Kyoda, Yosuke Iwadate, Eri Shishido, Keisuke Ikeda, Nana Kinoshita, Yasuki Matsuda, Sakiko Otani, Ryo Kameda, Kenta Matoba, Miwa Nonaka, Mika Maeda, Yuji Kumagai, Junya Ako, Masayoshi Shichiri, Katsuhiko Naoki, Masato Katagiri, Masashi Takaso, Masatsugu Iwamura, Kazuhiko Katayama, Takeshi Miyatsuka, Yasushi Orihashi, Kunihiro Yamaoka, for the CORVETTE-01 Study Group

**Affiliations:** ^1^Department of Rheumatology and Infectious Diseases, Kitasato University School of Medicine, Kanagawa, Japan; ^2^Department of Respiratory Medicine, Shonan Fujisawa Tokushukai Hospital, Kanagawa, Japan; ^3^Department of Respiratory Medicine, Tokyo Metropolitan Police Hospital, Tokyo, Japan; ^4^Department of Cardiovascular Medicine, Kitasato University Hospital, Kanagawa, Japan; ^5^Department of Respiratory Medicine, Kitasato University Hospital, Kanagawa, Japan; ^6^Department of Respiratory Medicine, Tama-Nambu Chiiki Hospital, Tokyo, Japan; ^7^Department of Endocrinology and Metabolism, Kitasato University Hospital, Kanagawa, Japan; ^8^Global Clinical Research Coordinating Center, Kitasato University Hospital, Kanagawa, Japan; ^9^Laboratory of Clinical Pharmacoepidemiology and Research and Education Center for Clinical Pharmacy, School of Pharmacy, Kitasato University, Kanagawa, Japan; ^10^Clinical Trial Center, Kitasato University Hospital, Kanagawa, Japan; ^11^Department of Diabetes, Endocrinology and Metabolism, Tokyo Kyosai Hospital, Tokyo, Japan; ^12^Department of Medical Laboratory Sciences, Kitasato University School of Allied Health Sciences, Tokyo, Japan; ^13^Department of Orthopedic Surgery, Kitasato University Hospital, Kanagawa, Japan; ^14^Department of Urology, Kitasato University Hospital, Kanagawa, Japan; ^15^Department of Infection Control and Immunology, Ōmura Satoshi Memorial Institute, Tokyo, Japan; ^16^Division of Clinical Research, Kitasato University Hospital, Kanagawa, Japan

**Keywords:** double-blind, ivermectin, SARS-CoV-2 proliferation, RT-PCR test, Japan

## Abstract

**Background:**

To investigate whether ivermectin inhibits SARS-CoV-2 proliferation in patients with mild-to-moderate COVID-19 using time to a negative COVID-19 reverse transcription-polymerase chain reaction (RT-PCR) test.

**Methods:**

CORVETTE-01 was a double-blind, randomized, placebo-controlled study (August 2020–October 2021) conducted in Japan. Overall, 248 patients diagnosed with COVID-19 using RT-PCR were assessed for eligibility. A single oral dose of ivermectin (200  μg/kg) or placebo was administered under fasting. The primary outcome was time to a negative COVID-19 RT-PCR test result for SARS-CoV-2 nucleic acid, assessed using stratified log-rank test and Cox regression models.

**Results:**

Overall, 112 and 109 patients were randomized to ivermectin and placebo, respectively; 106 patients from each group were included in the full analysis set (male [%], mean age: 68.9%, 47.9 years [ivermectin]; 62.3%, 47.5 years [placebo]). No significant difference was observed in the occurrence of negative RT-PCR tests between the groups (hazard ratio, 0.96; 95% confidence interval [CI] 0.70–1.32; *p* = 0.785). Median (95% CI) time to a negative RT-PCR test was 14.0 (13.0–16.0) and 14.0 (12.0–16.0) days for ivermectin and placebo, respectively; 82.1% and 84% of patients achieved negative RT-PCR tests, respectively.

**Conclusion:**

In patients with COVID-19, single-dose ivermectin was ineffective in decreasing the time to a negative RT-PCR test.

**Clinical Trial Registration:**

ClinicalTrials.gov, NCT04703205.

## 1. Introduction

SARS-CoV-2 is a highly transmissible pathogenic coronavirus causing COVID-19 (1). Novel therapeutic agents approved for COVID-19 have demonstrated efficacy, especially for early disease (2); however, they pose a huge economic burden on overstretched healthcare systems (3). Drug repurposing, an efficient strategy for identifying rapid treatment solutions by reducing developmental costs owing to preexisting data, could increase drug affordability and ensure equitable and wider drug distribution (3).

Ivermectin, a broad-spectrum antiparasitic agent from the ivermectin family with a long history of safety (4), has been widely used over two decades in humans, pets, and livestock (5). An *in vitro* study demonstrated that SARS-CoV-2 viral RNA was reduced with ivermectin 5 μM versus control (6). This ivermectin concentration is unlikely to be achieved in human lungs or plasma with routine antiparasitic doses (150–400 μg/kg) (7). However, at routine doses, ivermectin could possibly demonstrate additional immunomodulatory effects *in vivo* through its metabolites, potentially concentrated in the lung tissue (8–10).

Several real-world observational studies of ivermectin in COVID-19 have been conducted, but they have demonstrated conflicting efficacy results (11–14), raising a worldwide debate. Of the randomized controlled trials (RCTs) that demonstrated favorable efficacy, some were fraudulent or of poor quality with a high bias risk (15, 16). A recently published open-label, clinical study showed that ivermectin plus standard of care could not reduce the proportion of high-risk patients progressing to severe disease (17). We conducted an RCT, CORVETTE-01, which used the time to a negative reverse transcription–polymerase chain reaction (RT-PCR) test to investigate whether a single ivermectin dose (200 μg/kg) without any concomitant treatment in patients with mild-to-moderate COVID-19 could inhibit SARS-CoV-2 proliferation.

## 2. Methods

### 2.1. Study design

This was a multicenter, double-blind, placebo-controlled, randomized study conducted in Japan from August 2020 to October 2021 (ClinicalTrials.gov: NCT04703205). Eligible patients were randomized 1:1 to ivermectin or placebo (see Supplementary Figure S1) using dynamic allocation with the minimization method, with age (<65 years/≥65 years) and presence/absence of chronic obstructive pulmonary disease (COPD), diabetes mellitus, and pneumonia as adjustment factors.

Both patients and investigators were blinded to the study drug. Ivermectin and placebo, indistinguishable by appearance, were placed in aluminum bags and packaged in individual boxes. Allocation was centrally managed at Kitasato University Hospital using interactive web response systems. The investigator administered the study drug to patients according to the drug ID documented on the electronic case report form during allocation. To ensure blinding during administration of the study drug, patients wore an eye mask and were assisted by an unblinded collaborator.

The study was conducted in compliance with the ethical principles based on the Declaration of Helsinki and all applicable regulatory requirements and was approved by the institutional review board. All patients provided written informed consent. The Consolidated Standards of Reporting Trials (CONSORT) guidelines for RCTs were followed for reporting the study (18).

### 2.2. Patients

Patients aged ≥20 years who were diagnosed with COVID-19 (including asymptomatic cases) using an RT-PCR test within 3 days before eligibility assessment, reported room air oxygen saturation (SpO_2_) of ≥95%, weighed ≥40 kg at eligibility assessment, and could provide written informed consent were included in the study. Patients who received prohibited concomitant medications within the past month (past 6 months for biologics) or required prohibited concomitant medications (see Supplementary Table S1) during the study period or those scheduled for SARS-CoV-2 vaccination between the date of informed consent and the completion of follow-up were excluded from the study (see Supplementary Appendix 1). The rationale for excluding patients with scheduled SARS-CoV-2 vaccination was attributed to the uncertainty of the optimal timing of vaccination following COVID-19 and the potential challenge of distinguishing adverse reactions due to vaccination from those due to ivermectin.

### 2.3. Treatment

Ivermectin was purchased from MSD. For each patient, 10 tablets were prepared, and five of them were individually sealed in an aluminum package to ensure that the contents remained opaque. Patients wore an eye mask and received the dose according to their body weight, with the assistance of a nurse or a pharmacist (Supplementary Appendix 3). Patients received a single oral dose of either ivermectin (200 μg/kg) or placebo on an empty stomach (fasting) on day 1. During the observation period, treatment for preexisting underlying diseases and adverse events (AEs) unrelated to COVID-19 and symptomatic treatment for COVID-19 were permitted, but concomitant use of either COVID-19 medications or drugs with potential efficacy for COVID-19 (see Supplementary Table S1) was prohibited.

### 2.4. Study outcomes

The primary outcome was the time to a negative COVID-19 RT-PCR test. Nasopharyngeal swab specimens were obtained and tested for SARS-CoV-2 nucleic acid detection (positive or negative) at enrollment; on days 3, 5, 7, 9, 11, 13, and 15. Patients who did not test negative for SARS-CoV-2 on RT-PCR at the protocol-defined test visits were continued to be tested until a negative result occurred at bidaily intervals for up to a maximum of 45 days.

Secondary outcomes included the proportion of patients with a change in disease status between enrollment and day 15 (see Supplementary Appendix 2), with onset of pneumonia on day 15 and in the survival follow-up period, and requiring oxygen support; the proportion of patients and number of days on mechanical ventilation; the pattern of body temperature until day 15; the proportion of patients with a negative COVID-19 RT-PCR test until day 15; all deaths until the end of follow-up; the proportion of patients requiring, and number of days on, rescue treatment until day 15; the proportion of patients and number of days with severe arteriovenous thrombosis, including cerebral infarction and pulmonary thrombosis; the number of days required for the normalization of SpO_2_ in room air (SpO_2_ ≥95% lasting 24 h) when oxygen support was required; and the proportion of patients whose clinical status improved from enrollment by more than one stage from stage 3 of the 7-point ordinal scale on day 15.

AEs including subjective and objective findings at presentation and change in vital signs (blood pressure, pulse rate, and temperature) and AEs observed in laboratory parameters were assessed. Subjective findings were collected using an electronic device (see Supplementary Appendix 3).

### 2.5. Statistical analysis

The target sample size was set at 240 patients to achieve a significance level of 5% and a power of ≥80% in a two-sided log-rank test and considering that a few patients would be unevaluable. It was estimated that 192 events would be required among 210 patients in the two treatment groups over an observation period of 45 days, assuming that the median time to achieve a negative COVID-19 RT-PCR was 15 days with placebo and 10 days with ivermectin.

The full analysis set included all enrolled patients evaluated for efficacy at least once and excluded those who were ineligible after enrollment or did not receive the study drug. The safety analysis set included all enrolled patients, except those who did not receive the study drug.

Categorical variables were expressed as the number and percentage of patients and continuous variables as mean (standard deviation [SD]) or median (range). The Kaplan–Meier method was used to summarize the number of events and time to a negative RT-PCR test. The number of patients with a negative RT-PCR test and point estimates and confidence intervals (CIs) of the median time to a negative RT-PCR test were calculated. CIs were estimated using the Brookmeyer and Crowley method (19). Data for patients with rescue treatment were censored uniformly at 15 days. Patients who were judged by the physician to have difficulty in continuing the study due to worsening of symptoms (without rescue treatment) were treated in the same manner as patients receiving rescue treatment. In case of death, the patient was treated as not having a negative RT-PCR test until 45 days (censored at 45 days). To compare survival (time to a negative RT-PCR test) between the treatment groups, stratified log-rank tests were performed using the allocation adjustment factors as stratification factors between the groups. To quantitatively assess the treatment effect, the point estimates and CIs of adjusted hazard ratios (HRs) were calculated using stratified Cox regression models, with the allocation group as the explanatory variable and the allocation adjustment factors as stratification factors.

Sensitivity analysis for the primary outcome was performed by censoring 45-day data of patients who received rescue treatment and those excluded at the investigator’s discretion as being unable to continue treatment owing to worsening of symptoms. Exploratory analysis was conducted to assess the time to a negative RT-PCR test using survival analysis in subgroups stratified by age (<65 years/≥65 years) and presence/absence of COPD, diabetes mellitus, pneumonia, and smoking history. For secondary outcomes, the Cochran–Mantel–Haenszel test with the allocation adjustment factors as stratification factors was used to calculate adjusted odds ratios (ORs) and their 95% CIs to compare the differences between the ivermectin and placebo groups. Missing data were not imputed; however, no patient was excluded due to missing data.

The number of patients with AEs was summarized using system organ class (SOC) and preferred term using the Japanese translation of the Medical Dictionary for Regulatory Activities. AEs were listed by severity (Common Terminology Criteria for Adverse Events, version 5.0 grade).

Statistical analysis was performed using SAS (SAS Institute, Cary, NC, United States) version 9.4 (or later).

## 3. Results

### 3.1. Patient disposition

**Figure 1:**
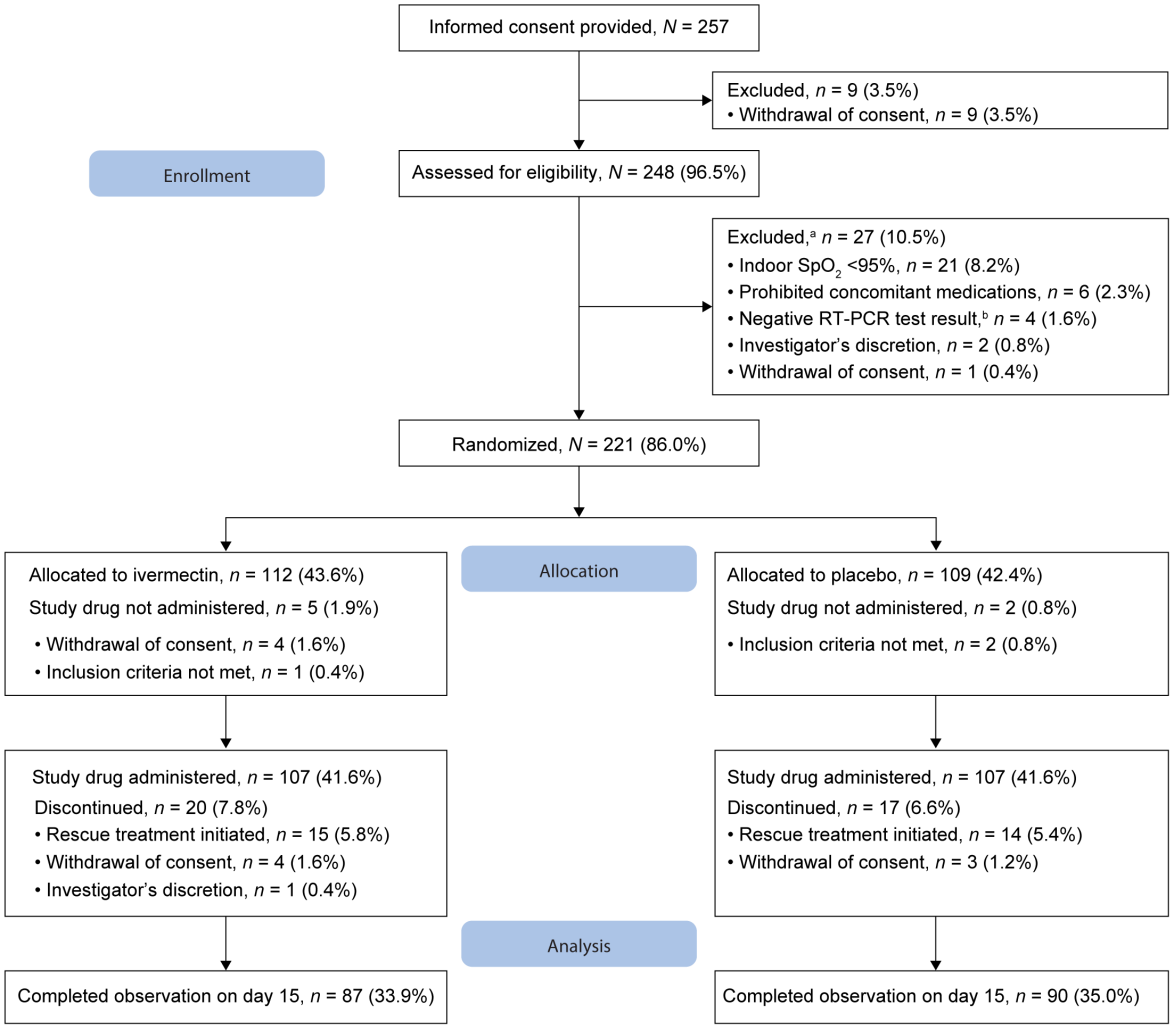
Patient disposition. ^a^Few patients met multiple criteria for exclusion. ^b^Within 3 days before eligibility assessment. RT-PCR, reverse transcription–polymerase chain reaction; SpO_2_, oxygen saturation.

Of the 257 patients who provided informed consent, nine (3.5%) withdrew consent and 248 were assessed for eligibility (Figure 1). Furthermore, 27 patients were excluded before randomization. As the number of infected patients decreased considerably in Japan, the study was discontinued before the targeted sample size of 240 patients could be achieved. Overall, 221 patients were randomized 1:1 to ivermectin (*n* = 112, 43.6%) and placebo (*n* = 109, 42.4%); 107 patients in each treatment group received the study drug (Figure 1). The full analysis set included 106 patients in each treatment group following the exclusion of one patient each owing to the use of prohibited concomitant drugs within a month before study drug administration. By day 15, 87 and 90 patients in the ivermectin and placebo groups, respectively, completed the observation period. Overall, 25 patients in the ivermectin group and 19 in the placebo group discontinued the study (Figure 1). No deaths were reported in either treatment group.

### 3.2. Patient demographics and baseline characteristics

Demographics and baseline characteristics were generally well balanced between the two treatment groups, with mean (SD) age (years) of 47.9 (15.1) and 47.5 (15.0) in the ivermectin and placebo groups, respectively. Most patients were male (ivermectin, 68.9%; placebo, 62.3%) and reported comorbidities at baseline (ivermectin, 72.6%; placebo, 64.2%). Over 70% of patients in both treatment groups had pneumonia at baseline (ivermectin, 73.6%; placebo, 72.6%). Nine patients (8.5%) and seven patients (6.6%) in the ivermectin group and the placebo group, respectively, had prior SARS-CoV-2 vaccination.

The mean (SD) duration from the occurrence of a positive RT-PCR test to the administration of the study drug in the ivermectin and placebo groups was 2.7 (1.1) days and 2.7 (1.1) days, respectively; the corresponding mean (SD) duration from the first onset of a subjective symptom/first positive RT-PCR test to the administration of the study drug was 6.6 (2.6) days and 6.9 (2.8) days (Table 1). The treatment compliance rate was 100% in both treatment groups. During the study period in Japan, the Alpha (PANGO lineage; B.1.1.7) and Delta (AY.29) variants were predominant (20). Owing to the rapid emergence and spread of the new variant, conducting uniform testing across different facilities was challenging, particularly as the Alpha variant was being replaced by the new variant. Therefore, an analysis of the variants was not performed.

**Table 1:** Patient demographic and baseline characteristics (full analysis set).

Variable	Ivermectin (*N* = 106)	Placebo (*N* = 106)
Age, years		
Mean (SD)	47.9 (15.1)	47.5 (15.0)
Categories, no. (%)		
<65	89 (84.0)	91 (85.8)
≥65	17 (16.0)	15 (14.2)
Sex, male, no. (%)	73 (68.9)	66 (62.3)
BMI, mean (SD), kg/m^2^	25.1 (5.3)	24.0 (3.8)
Drug allergy, yes, no. (%)	7 (6.6)	4 (3.8)
Smoking status, no. (%)		
Yes (current smoker)	33 (31.1)	28 (26.4)
Yes (ex-smoker)	22 (20.8)	28 (26.4)
No	51 (48.1)	50 (47.2)
Comorbidities, yes, no. (%)	77 (72.6)	68 (64.2)
Chronic obstructive pulmonary disease, yes, no. (%)	2 (1.9)	2 (1.9)
Diabetes mellitus, yes, no. (%)	14 (13.2)	15 (14.2)
Pneumonia, yes, no. (%)	78 (73.6)	77 (72.6)
Medical history of other disorders, yes, no. (%)	42 (39.6)	41 (38.7)
Days from a positive RT-PCR test to study drug administration		
Mean (SD)	2.7 (1.1)	2.7 (1.1)
Median (range)	2.0 (1–6)	2.0 (1–6)
Days from the first onset of subjective symptom or first positive RT-PCR test to study drug administration		
Mean (SD)	6.6 (2.6)	6.9 (2.8)
Median (range)	6.0 (1–15)	7.0 (2–20)
SARS-CoV-2 vaccination, no. (%)	9 (8.5)	7 (6.6)

BMI, body mass index; RT-PCR, reverse transcription–polymerase chain reaction; SD, standard deviation.

### 3.3. Efficacy

**Figure 2:**
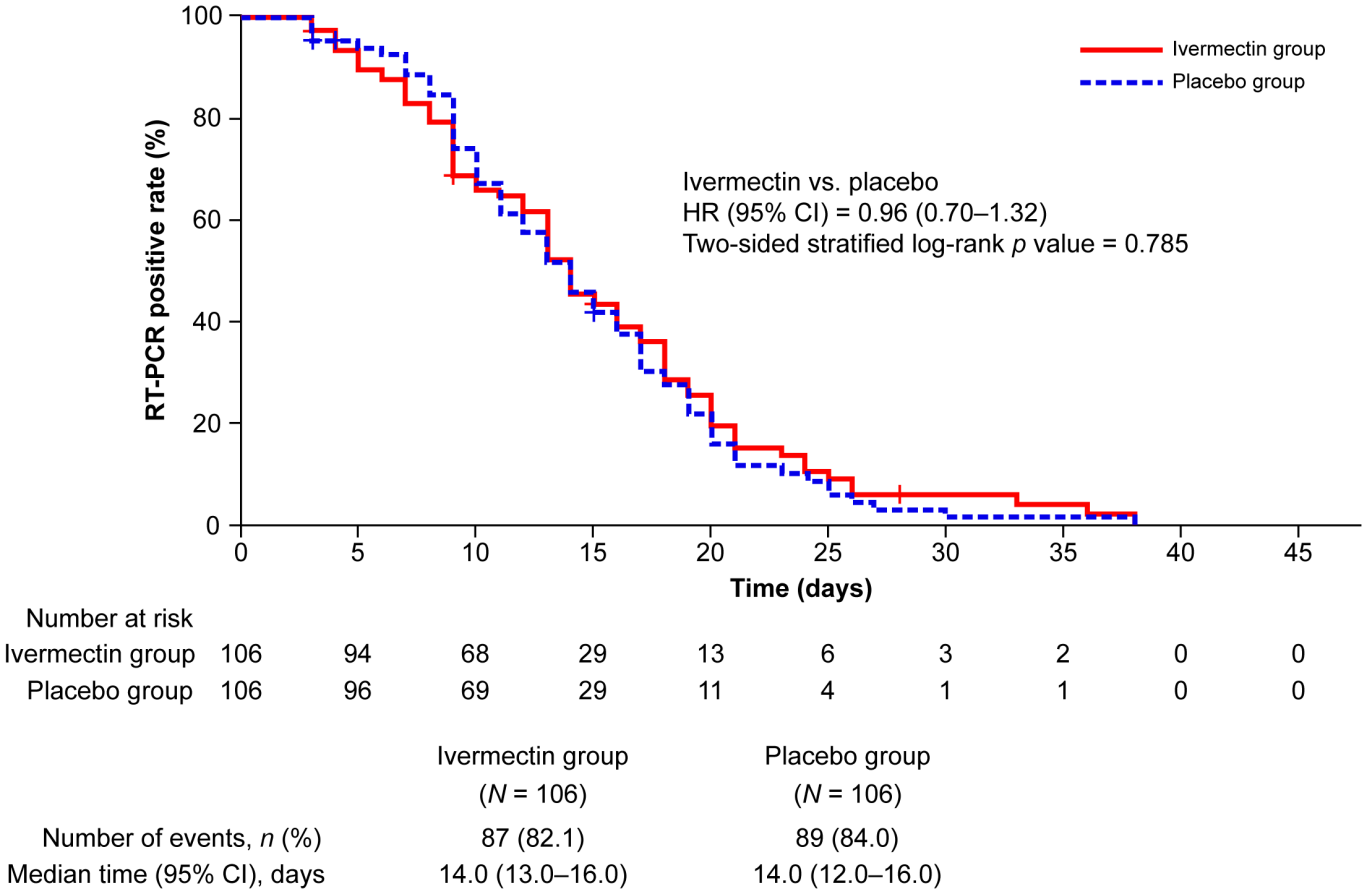
Kaplan–Meier plot for time to a negative RT-PCR test. “Number at risk” for each follow-up period denotes the number of patients who were traceable and confirmed to have an RT-PCR-positive result. CI, confidence interval; HR, hazard ratio; RT-PCR, reverse transcription–polymerase chain reaction.

One patient in each treatment group was excluded from the efficacy analysis because of their use of a prohibited medication within 1 month before the administration of the study drug. The survival analysis showed no significant difference in the occurrence of negative RT-PCR test results between the ivermectin and placebo groups (HR, 0.96; 95% CI, 0.70–1.32; *p* = 0.785; HR <1 favors placebo, whereas HR >1 favors ivermectin). The median (95% CI) time to a negative RT-PCR test was 14.0 (13.0–16.0) days and 14.0 (12.0–16.0) days in the ivermectin and placebo groups, respectively. Of 106 patients in each treatment group, 82.1% (*n* = 87) and 84% (*n* = 89) of patients achieved a negative RT-PCR test result with ivermectin and placebo, respectively, over the follow-up period for up to 45 days (Figure 2).

**Figure 3:**
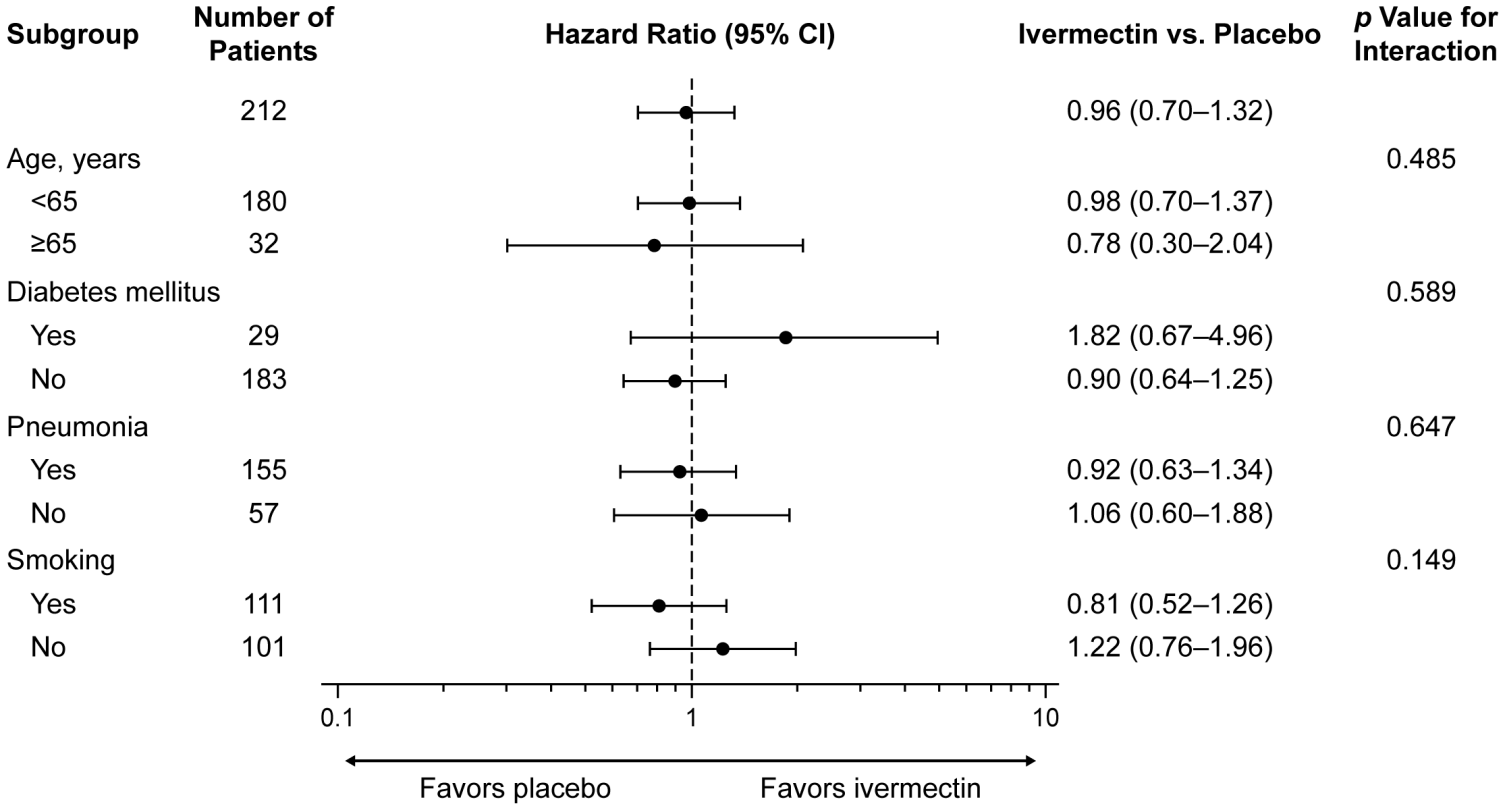
Subgroup analysis for time to a negative RT-PCR test. CI, confidence interval; RT-PCR, reverse transcription–polymerase chain reaction.

The sensitivity analysis yielded consistent results. The occurrence of a negative PCR test by age <65 years versus ≥65 years, with/without a history of COPD, diabetes mellitus, pneumonia, and smoking, and the interaction effect between treatment groups and subgroups was not significantly different (Figure 3; see Supplementary Table S2).

From enrollment to day 15, a similar proportion of patients in the ivermectin (17.9%) and placebo (21.7%) groups showed disease worsening (OR, 0.77; 95% CI, 0.38–1.54; *p* = 0.462; see Supplementary Table S3). No notable difference was observed in the mean (SD) maximum daily temperatures from day 1 to day 15 in patients receiving ivermectin or placebo (see Supplementary Figure S2).

### 3.4. Safety

Overall, 29 patients (27.1%) in the ivermectin group and 28 (26.2%) in the placebo group reported AEs (Table 2). Six patients (5.6%) in the ivermectin group and five (4.7%) in the placebo group reported treatment-emergent AEs (TEAEs). Grade ≥3 TEAEs occurred in one (0.9%) patient in the ivermectin group and two (1.9%) patients in the placebo group. The most common TEAEs reported by SOC were gastrointestinal disorders and hepatobiliary system disorders (*n* = 2, 1.9% each) in the ivermectin group and hepatobiliary system disorders (*n* = 2, 1.9%) in the placebo group. No patient reported a TEAE that led to study discontinuation.

**Table 2:** Safety outcomes (safety analysis set).

AEs	Ivermectin *N* = 107		Placebo *N* = 107	
All AEs, no. (%)	29 (27.1)		28 (26.2)	
AEs by severity (CTCAE grade), no. (%)
Grade 1	19 (17.8)		17 (15.9)	
Grade 2	9 (8.4)		9 (8.4)	
Grade 3	1 (0.9)		1 (0.9)	
Grade 4	0		1 (0.9)	
Serious AEs, no. (%)	1 (0.9)		1 (0.9)	
AEs that led to study discontinuation, no. (%)	0		0	
All TEAEs, no. (%)	6 (5.6)		5 (4.7)	
TEAEs by severity (CTCAE grade), no. (%)
Grade 1	3 (2.8)		3 (2.8)	
Grade 2	2 (1.9)		0	
Grade 3	1 (0.9)		1 (0.9)	
Grade 4	0		1 (0.9)	
Serious TEAEs, no. (%)	0		1 (0.9)	
TEAEs that led to study discontinuation, no. (%)	0		0	
TEAE by SOC and PT	Patients, No. (%)	Events, No.	Patients, No. (%)	Events, No.
Gastrointestinal disorders	2 (1.9)	2	1 (0.9)	1
Diarrhea	2 (1.9)	2	0	0
Loose stools	0	0	1 (0.9)	1
Hepatobiliary system disorders	2 (1.9)	2	2 (1.9)	2
Hepatic function abnormal	1 (0.9)	1	2 (1.9)	2
Liver disorders	1 (0.9)	1	0	0
Musculoskeletal and connective tissue disorders	0	0	1 (0.9)	1
Myalgia	0	0	1 (0.9)	1
Skin and subcutaneous tissue disorders	1 (0.9)	1	1 (0.9)	1
Rash	1 (0.9)	1	1 (0.9)	1
Investigations	1 (0.9)	1	1 (0.9)	2
Aspartate aminotransferase increased	0	0	1 (0.9)	1
Alanine aminotransferase increased	0	0	1 (0.9)	1
Blood creatine phosphokinase increased	1 (0.9)	1	0	0
TEAE grade ≥ 3 by PT	Patients, No. (%)	Events, No.	Patients, No. (%)	Events, No.
Hepatic function abnormal	0	0	2 (1.9)^a^	2
Blood creatine phosphokinase increased	1 (0.9)	1	0	0

AE, adverse event; CTCAE, Common Terminology Criteria for Adverse Events; PT, preferred term; SOC, system organ class; TEAE, treatment-emergent adverse event.

a One grade 3 and one grade 4.

One patient (0.9%) in the ivermectin group who reported a serious AE of the acute abdomen of grade 2 severity recovered. One patient reported blood creatine phosphokinase increased of grade ≥3 severity in the ivermectin group on day 15, and this TEAE was associated with the study drug; the patient did not improve, but the event was considered mild, and no follow-up was required based on the general condition of the patient. Two patients in the placebo group reported hepatic function abnormal of grade ≥3 severity, one of which was serious and of grade 4 severity; the condition of both patients improved (Table 2).

The mean (SD) changes in systolic/diastolic blood pressures and pulse rate from baseline to day 15 were 0.3 (17.68) mm Hg/−2.5 (12.58) mm Hg and 81.0 (11.80) min^−1^, respectively, in the ivermectin group (see Supplementary Table S4). The proportions of patients with clinical findings of sore throat, nasal discharge, cough, sputum, dyspnea, headache, nausea/vomiting, abdominal pain, diarrhea, myalgia, and arthralgia in both treatment groups are shown in Supplementary Figure S3. The severity scores for malaise, chest pain, diarrhea, and anosmia are reported in Supplementary Figure S4.

## 4. Discussion

This is the first multicenter, double-blind, placebo-controlled RCT in Japan that investigated the efficacy and safety of a single intervention with ivermectin monotherapy in patients with COVID-19. The mean time from a positive RT-PCR test to study drug administration (2.7 days) and the treatment compliance rate (100%) were similar for both treatment groups. No significant difference in the time to a negative RT-PCR test was observed between ivermectin-and placebo-treated patients with mild-to-moderate COVID-19.

In our study, the median duration to a negative RT-PCR test result was 14.0 days, and a similar proportion (⁓82%) of patients achieved a negative RT-PCR test result beyond the 15-day observation period, up to the end of the follow-up period. This result was observed with both ivermectin and placebo without any concomitant medication with potential efficacy for COVID-19. These results remained consistent in the sensitivity analysis and were similar to those from other RCTs with different dosages of ivermectin with/without concomitant treatments. In line with our study results with single-dose ivermectin 12 mg over a 15-day follow-up, the difference in the proportion of inpatients achieving a negative RT-PCR on days 5–6 with ivermectin versus placebo was nonsignificant with single doses of 12 mg/24 mg (8) or a 2-day course of 12-mg dose (21) in other double-blind RCTs. Similarly, a 3-day course of ivermectin 400 μg/kg (vs. placebo) demonstrated no significant difference in viral clearance on day 7 in high-risk symptomatic COVID-19 patients (22). Furthermore, no difference in the proportion of patients with a positive RT-PCR test on day 7 was observed with a single dose of ivermectin 400 μg/kg (vs. placebo) in patients with non-severe COVID-19 in another double-blind RCT (23). Thus, most RCTs demonstrated no significant increase in the proportion of patients achieving a negative RT-PCR test or viral clearance with standard doses of ivermectin for 1–3 days. However, a 5-day course of once-daily ivermectin 12 mg reduced the time to a negative RT-PCR test both versus placebo and with concomitant doxycycline 200 mg; the reduction was significant only versus placebo (9.7 days vs. 12.7 days; *p* = 0.005) (24).

The disparity in the efficacy of ivermectin in patients with COVID-19 observed among different trials could be attributed to differences in patient characteristics, ivermectin dosage, exposures, and measured outcomes and to unidentified confounders. Studies that showed favorable outcomes with ivermectin for COVID-19 generally used a higher dose (25) or multiday dosing (24), which could explain the lack of difference when compared with placebo observed in our study where ivermectin was used at the approved antiparasitic dose of 200 μg/kg. The antiviral activity of ivermectin in COVID-19 has been suggested to be dose dependent (26) and concentration dependent (27). Modeling simulation studies have predicted an unlikely probability of lung ivermectin concentration attaining the desired half-maximal inhibitory concentration of 2 μM following a single, approved, oral dose or even a dose 10 times higher (28, 29); however, lung tissue concentrations of ivermectin remain unsubstantiated from those in human studies. Furthermore, data to support a recommendation for ivermectin use in higher-than-approved doses are limited, although the safety profile of high (>400 μg/kg) and standard (≤400 μg/kg) doses is reportedly comparable (30). Moreover, doses up to 10 times the approved doses and more frequent dosing regimens are reportedly well tolerated (31). In our study, the incidence of TEAEs was low with ivermectin 200 μg/kg, confirming the safety of the standard dose in patients with COVID-19. Because efficacy was lacking at the standard dose, higher doses may be recommended, although the I-TECH trial has raised safety concerns (17). Therefore, different treatment protocols employing varying dosing regimens and drug delivery systems such as inhalers to achieve higher and more targeted, localized concentrations in the lungs while minimizing systemic exposure (32) can further elucidate the potential role of ivermectin in COVID-19 treatment.

In Japan, 6,832,377 confirmed cases of COVID-19 were reported until March 2022 (33), which is relatively lower than the number of cases reported worldwide. Furthermore, despite a large elderly population, Japan has a relatively lower number of deaths due to COVID-19 (28,456 deaths) (33), and no deaths were reported in our study. Thus, ethnicity may potentially impact COVID-19 characteristics and treatment response to various drugs in Japanese patients.

A recent report (16) that highlighted the role of fraudulent studies and studies with questionable evidence in causing a bias in favor of ivermectin reiterates the importance of scientific rigor in achieving unbiased, accurate, and robust outcomes. Our double-blind, placebo-controlled, randomized study with a reasonable sample size was carefully designed and conducted across multiple hospitals to minimize the risk of bias. Moreover, a detailed safety analysis was conducted, and pneumonia was assessed using computed tomography images. However, the results may not be generalizable because the study population comprised patients only of Japanese ethnicity. Furthermore, ivermectin has a plasma half-life of ⁓18 h, suggesting that multiple doses of ivermectin may be beneficial; however, only a single dose of ivermectin at the fasting state, as approved in Japan, was used in our study. The fasting state could have also contributed to our results, which is known to result in a lower blood drug concentration than that after postprandial administration.

## 5. Conclusion

The current study showed that, compared with placebo, single-dose ivermectin treatment was well tolerated but did not lead to a decrease in the time to a negative RT-PCR test for patients with mild-to-moderate COVID-19.

## Data availability statement

The original contributions presented in the study are included in the article/Supplementary material, further inquiries can be directed to the corresponding author.

## Ethics statement

The studies involving human participants were reviewed and approved by the IRB of Kitasato University Shirokane Campus, affiliation: Kitasato University. The patients/participants provided their written informed consent to participate in this study.

## Author contributions

KY contributed towards conceptualization and drafting the work. YK contributed towards conceptualization, drafting the work, and revising it critically for important intellectual content. MS, TM, JA, KN, MI, MT, MM, MK, and KK contributed towards conceptualization, drafting the work, and revising it critically for important intellectual content. MH, HA, MN, KI, ES, YI, NK, SK, SO, RK, KM, and YM contributed towards acquisition, analysis, or interpretation of data for the work, drafting the work, and revising it critically for important intellectual content. TW contributed towards conceptualization, acquisition, analysis, or interpretation of data for the work, and drafting the work. YO contributed towards the data analysis, drafting the work, and revising it critically for important intellectual content. All authors provide approval for publication of the content and agree to be accountable for all aspects of the work in ensuring that questions related to the accuracy or integrity of any part of the work are appropriately investigated and resolved.

## Funding

This work was supported by public funding [AMED grant number: JP20fk0108159] from the “Research Program on Creation of Research Base for Emerging and Re-emerging Infectious Diseases: Development of Therapeutic Drugs for the Novel Coronavirus Disease (Covid-19)” of the Japan Agency for Medical Research and Development and “Kitasato Funding Project for Actions Against COVID-19” of the Kitasato Institute.

## Conflict of interest

The authors declare that the research was conducted in the absence of any commercial or financial relationships that could be construed as a potential conflict of interest.

A reviewer of this manuscript, Devan Mehrotra, is a biostatistician at Merck & Co., Inc., known as MSD outside the United States and Canada. The pharmaceutical ivermectin studied in this manuscript is manufactured and marketed by Merck & Co., Inc. in the United States under the brand name STROMECTOL®. After investigation, the journal has no reason to believe that the scientific conclusions of the article are affected in any way.

## Publisher’s note

All claims expressed in this article are solely those of the authors and do not necessarily represent those of their affiliated organizations, or those of the publisher, the editors and the reviewers. Any product that may be evaluated in this article, or claim that may be made by its manufacturer, is not guaranteed or endorsed by the publisher.
