# Erratum: Efficacy and safety of single-dose ivermectin in mild-to-moderate COVID-19: the double-blind, randomized, placebo-controlled CORVETTE-01 trial

**DOI:** 10.3389/fmed.2023.1289307

**Published:** 2023-09-19

**Authors:** Frontiers Production Office

**Affiliations:** Frontiers Media SA, Lausanne, Switzerland

**Keywords:** double-blind, ivermectin, SARS-CoV-2 proliferation, RT-PCR test, Japan

Due to a production error, an incorrect Conflict of Interest statement was provided. The correct Conflict of Interest statement appears below. The publisher apologizes for this mistake.

“The authors declare that the research was conducted in the absence of any commercial or financial relationships that could be construed as a potential conflict of interest.

A reviewer of this manuscript, Devan Mehrotra, is a biostatistician at Merck & Co., Inc., known as MSD outside the United States and Canada. The pharmaceutical ivermectin studied in this manuscript is manufactured and marketed by Merck & Co., Inc. in the United States under the brand name STROMECTOL^®^. After investigation, the journal has no reason to believe that the scientific conclusions of the article are affected in any way.”

The original article has been updated.

