# Supplementary material for: Efficacy and safety of single-dose ivermectin in mild-to-moderate COVID-19: the double-blind, randomized, placebo-controlled CORVETTE-01 trial
Source: Front Med (Lausanne). 2023 May 22;10:1139046. doi: 10.3389/fmed.2023.1139046 (PMC10240959; doi:10.3389/fmed.2023.1139046)
Supplement: Supplementary file 1 [file Data_Sheet_1.docx]

**Supplementary Figure S1. Study design**


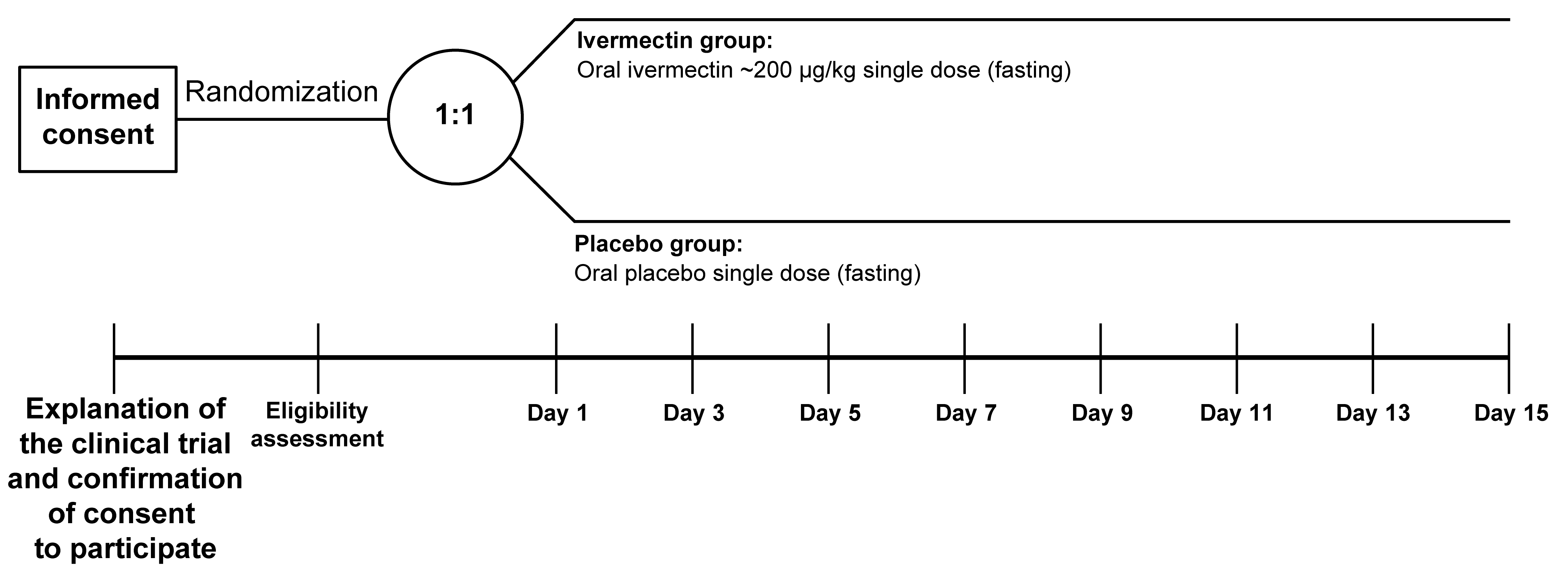


**Supplementary Figure S2. Body temperature (highest daily temperature for each patient) up to day 15**

Error bars represent standard deviation values for mean body temperature.

**Supplementary Figure S3. Distribution of clinical findings from baseline to day 15 (safety analysis set)**


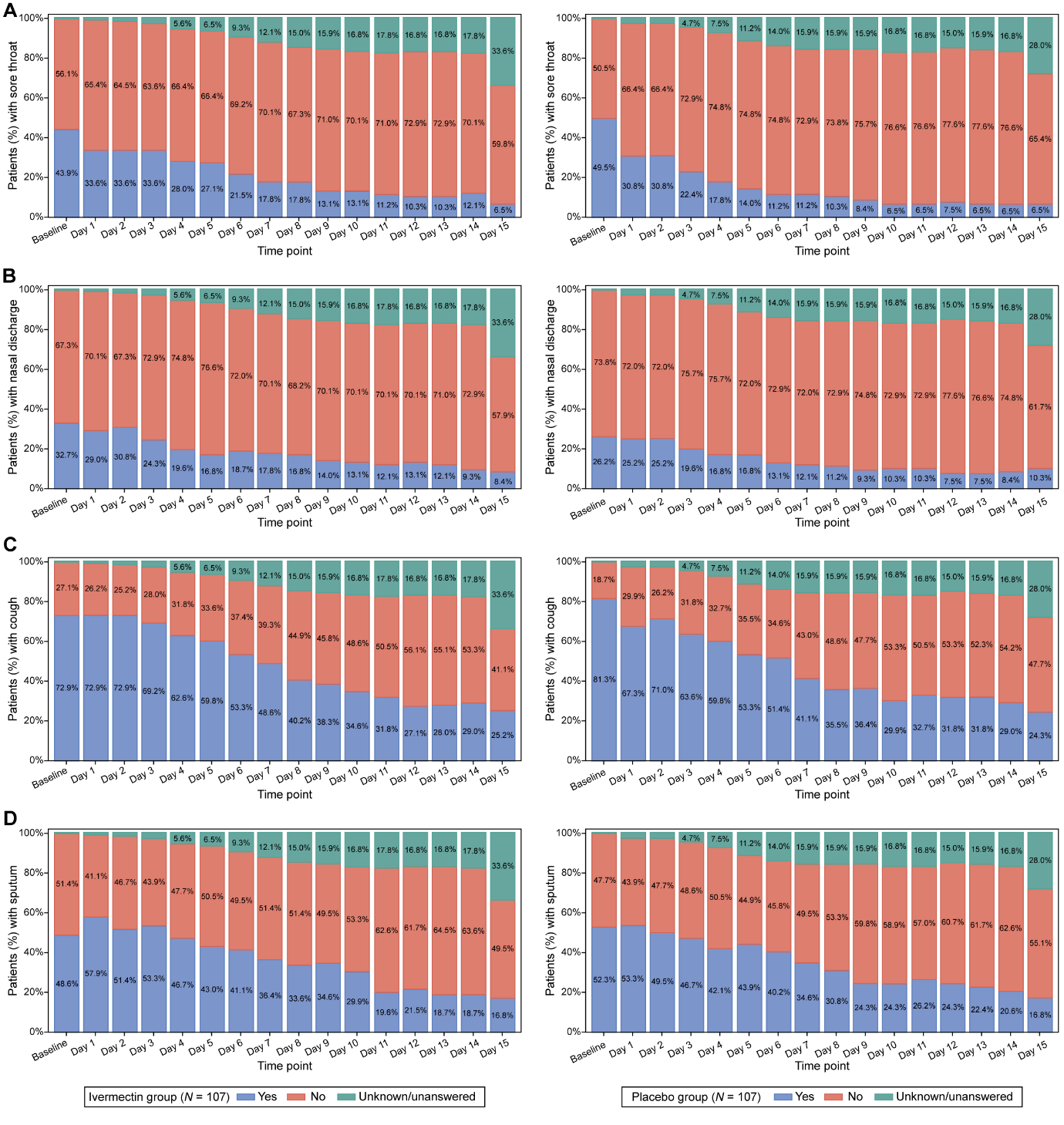


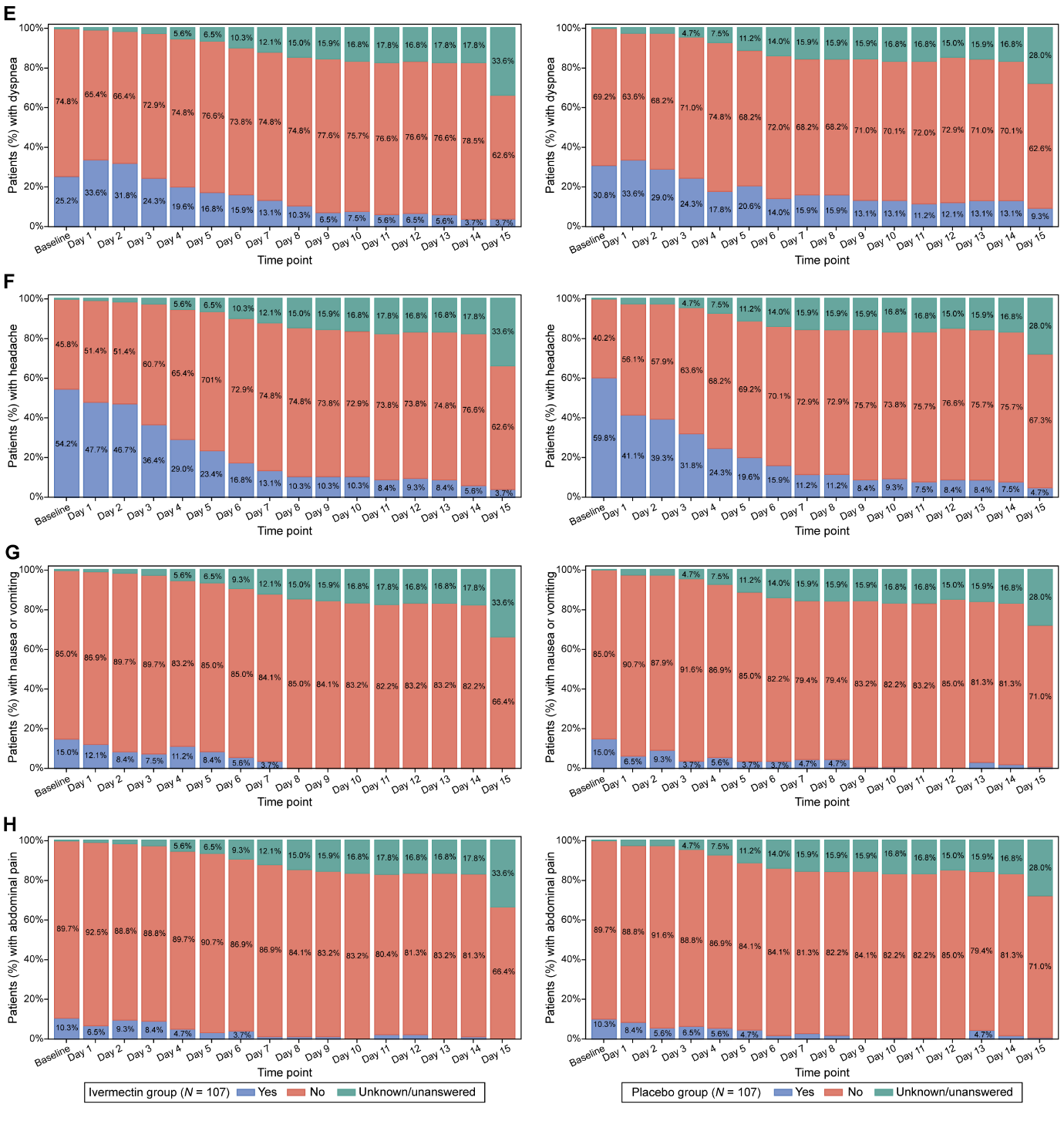


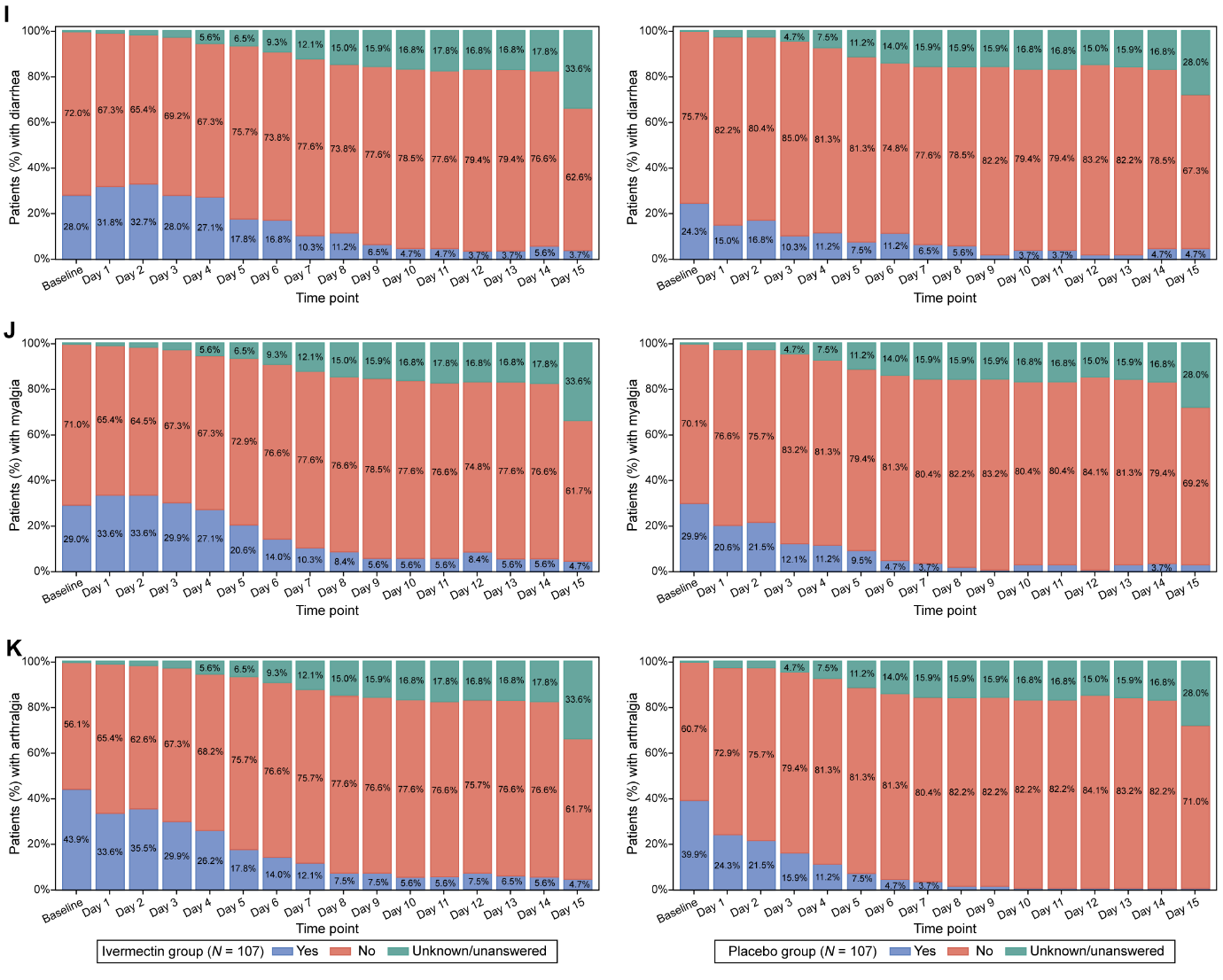


**Supplementary Figure S4. Severity scores for malaise/chest pain/dysgeusia/anosmia from baseline to day 15 (safety analysis set)**

**
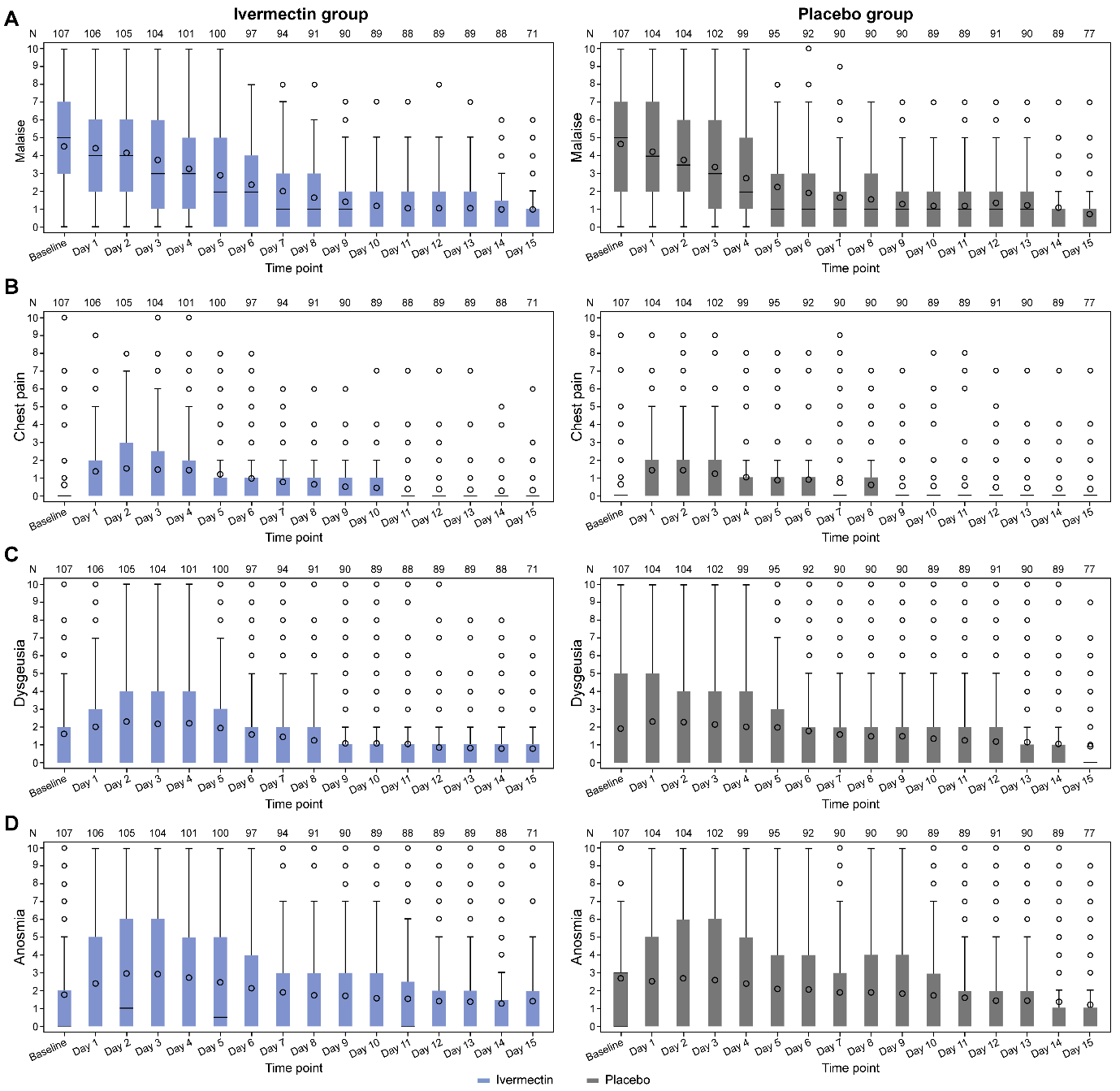
**

Dots represent outliers (1.5 times above the 75th percentile at the top and 1.5 times below the 25th percentile at the bottom).

Severity scores: 0 (no malaise/chest pain/dysgeusia/anosmia) to 10 (severe malaise/chest pain/dysgeusia/anosmia).

**Supplementary Table S1. List of prohibited concomitant drugs**

| Drug class | Generic name |
| --- | --- |
| Anti-HIV drug | Lopinavir  Ritonavir  Nelfinavir |
| Anti-ebolavirus drugs | Remdesivir |
| Anti-influenza drugs | Favipiravir |
| Antimalarials | Hydroxychloroquine |
| Anti-asthma drugs (inhaled corticosteroids) | Ciclesonide |
| Systemic corticosteroids (oral, injectable, and suppository) | Prednisolone  Methylprednisolone  Dexamethasone |
| Drugs for pancreatitis | Camostat  Nafamostat |
| Hepatitis C drugs | Interferon preparations  Ribavirin |
| Biological preparations Drugs for new coronavirus (antibody cocktail therapy) | Casirivimab  Imdevimab |
| Biological agents (humanized anti–IL-6 receptor monoclonal antibody) | Tocilizumab  Sarilumab |
| JAK inhibitors | Tofacitinib  Baricitinib |
| Macrolide antibiotics | Azithromycin |
| Drugs for gout and familial Mediterranean fever | Colchicine |

HIV, human immunodeficiency virus; IL, interleukin; JAK, Janus kinase.

**Supplementary Table S2. Subgroup analysis for time to a negative RT-PCR test**

| **Variable** | **Ivermectin** | **Placebo** |
| --- | --- | --- |
| Age (<65 y) | *n* = 89 | *n* = 91 |
| Number of events, No. (%) | 74 (83.1) | 78 (85.7) |
| Median time (95% CI), d | 13.0 (12.0–16.0) | 14.0 (11.0–16.0) |
| Age (≥65 y) | *n* = 17 | *n* = 15 |
| Number of events, No. (%) | 13 (76.5) | 11 (73.3) |
| Median time (95% CI), d | 18.0 (9.0–24.0) | 16.0 (9.0–20.0) |
| COPD (no) | *n* = 104 | *n* = 104 |
| Number of events, No. (%) | 85 (81.7) | 87 (83.7) |
| Median time (95% CI), d | 14 (13–16) | 14 (12–16) |
| Diabetes mellitus (yes) | *n* = 14 | *n* = 15 |
| Number of events, No. (%) | 12 (85.7) | 12 (80.0) |
| Median time (95% CI), d | 14.0 (9.0–24.0) | 16.0 (3.0–20.0) |
| Diabetes mellitus (no) | *n* = 92 | *n* = 91 |
| Number of events, No. (%) | 75 (81.5) | 77 (84.6) |
| Median time (95% CI), d | 14.0 (13.0–16.0) | 13.0 (11.0–15.0) |
| Pneumonia (yes) | *n* = 78 | *n* = 77 |
| Number of events, No. (%) | 61 (78.2) | 62 (80.5) |
| Median time (95% CI), d | 13.0 (13.0–16.0) | 14.0 (11.0–16.0) |
| Pneumonia (no) | *n* = 28 | *n* = 29 |
| Number of events, No. (%) | 26 (92.9) | 27 (93.1) |
| Median time (95% CI), d | 15.5 (8.0–19.0) | 14.0 (11.0–17.0) |
| History of smoking (yes: current smoker/ex-smoker) | *n* = 55 | *n* = 56 |
| Number of events, No. (%) | 44 (80.0) | 50 (89.3) |
| Median time (95% CI), d | 14.0 (13.0–18.0) | 13.0 (11.0–16.0) |
| History of smoking (no) | *n* = 51 | *n* = 50 |
| Number of events, No. (%) | 43 (84.3) | 39 (78.0) |
| Median time (95% CI), d | 13.0 (9.0–16.0) | 14.0 (11.0–18.0) |

CI, confidence interval; COPD, chronic obstructive pulmonary disease; RT-PCR, reverse transcription–polymerase chain reaction.

**Supplementary Table S3. Secondary outcomes**

|  | **Ivermectin** | **Placebo** | **Cochran-Mantel-Haenszel test** |  |
| --- | --- | --- | --- | --- |
| **Outcome** | ***n*/*N* (%)** | ***n*/*N* (%)** | **Adjusted OR (95% CI)** | ***p* value** |
| Worsening (up to day 15) | 19/106 (17.9) | 23/106 (21.7) | 0.77 (0.38–1.54) | 0.462 |
| Improvement (up to day 15) | 15/106 (14.2) | 18/106 (17.0) | 0.81 (0.36–1.80) | 0.608 |
| Onset of pneumonia^a^ | 7/28 (25.0) | 9/29 (31.0) | 0.65 (0.20–2.09) | 0.469 |
| Oxygen support | 22/106 (20.8) | 19/106 (17.9) | 1.18 (0.58–2.43) | 0.652 |
| RT-PCR negativity rate (up to day 15) | 59/106 (55.7) | 60/106 (56.6) | 0.97 (0.56–1.68) | 0.912 |
| Rescue treatment initiated (up to day 15) | 15/106 (14.2) | 14/106 (13.2) | 0.97 (0.43–2.19) | 0.942 |

^a^In patients with no pneumonia at the time of enrollment.

CI, confidence interval; OR, odds ratio; RT-PCR, reverse transcription–polymerase chain reaction.

**Supplementary Table S4. Change in vital signs (safety analysis set)**

| Outcome | Ivermectin (*N* = 107) | | Placebo  (*N* = 107) | |
| --- | --- | --- | --- | --- |
|  | **Absolute value** | **Change** | **Absolute value** | **Change** |
| Systolic blood pressure (mm Hg) | | | | |
| Baseline (randomization) |  |  |  |  |
| No. | 107 |  | 106 |  |
| Mean (SD) | 122.5 (17.80) |  | 119.6 (16.26) |  |
| Median | 123.0 |  | 119.5 |  |
| Min, max | 81, 202 |  | 91, 163 |  |
| Day 15 |  |  |  |  |
| No. | 87 | 87 | 90 | 89 |
| Mean (SD) | 123.1 (15.65) | 0.3 (17.68) | 119.9 (15.91) | 1.0 (16.82) |
| Median | 121.0 | 0.0 | 119.5 | 1.0 |
| Min, max | 81, 170 | −66, 55 | 85, 159 | −42, 60 |
| Diastolic blood pressure (mm Hg) | | | | |
| Baseline (randomization) |  |  |  |  |
| No. | 107 |  | 106 |  |
| Mean (SD) | 78.2 (12.23) |  | 76.4 (12.21) |  |
| Median | 79.0 |  | 76.0 |  |
| Min, max | 43, 104 |  | 53, 108 |  |
| Day 15 |  |  |  |  |
| No. | 87 | 87 | 90 | 89 |
| Mean (SD) | 76.0 (10.69) | −2.5 (12.58) | 72.6 (11.84) | −2.8 (13.10) |
| Median | 76.0 | −4.0 | 71.5 | −4.0 |
| Min, max | 53, 108 | −30, 39 | 48, 117 | −42, 38 |
| Pulse, min^−1^ |  |  |  |  |
| Baseline (randomization) |  |  |  |  |
| No. | 107 |  | 106 |  |
| Mean (SD) | 81.0 (11.80) |  | 80.7 (13.16) |  |
| Median | 80.0 |  | 79.5 |  |
| Min, max | 55, 109 |  | 55, 107 |  |
| Day 15 |  |  |  |  |
| No. | 87 | 87 | 90 | 89 |
| Mean (SD) | 79.7 (12.73) | −0.6 (13.08) | 78.1 (11.85) | −1.2 (11.44) |
| Median | 78.0 | −1.0 | 79.0 | −1.0 |
| Min, max | 56, 116 | −31, 28 | 56, 107 | −41, 27 |

max, maximum; min, minimum; SD, standard deviation.

**Supplementary Table S5. List of investigators**

| **Medical institution** | **Principal investigator** |
| --- | --- |
| Uji Tokushukai Medical Center | Jun Shinozuka |
| Kitasato University Medical Center | Yuki Bando |
| Kitasato University Kitasato Institute Hospital | Yusuke Suzuki |
| Tokyo Nishi Tokushukai Hospital | Hirokazu Iijima |
| Tomakomai City Hospital | Tetsuya Horita |
| Ogikubo Hospital | Susumu Fujii |
| National Hospital Organization Maizuru Medical Center | Hiroshi Komatsu |
| St. Luke's International Hospital | Nobuyoshi Mori |
| Shiga University of Medical Science Hospital | Yasuki Uchida |
| Sagamihara Kyodo Hospital | Michiko Yamamoto |
| Japanese Red Cross Narita Hospital | Ryota Hase |
| Tokyo Metropolitan Cancer and Infectious Diseases Center, Komagome Hospital | Kazuteru Ohashi |
| Tokyo Metropolitan Bokutoh Hospital | Fukumi Nakamura |
| Kizawa Memorial Hospital | Takuma Aoyama |
| Kawasaki Municipal Ida Hospital | Takahiro Suzuki |
| Hiroshima University Hospital | Hiroki Ohge |
| Kuramochi Clinic Interpark | Jin Kuramochi |
| Toranomon Hospital Kajigaya | Atsushi Wake |
| Keio University Hospital | Shotaro Chubachi |
| Okayama University Hospital | Hideharu Hagiya |
| Okayama Rosai Hospital | Nobukazu Fujimoto |
| Okayama City Hospital | Kazuya Kariyama |
| Fujimino Emergency Hospital | Akira Kano |

**Supplementary Text**

**Supplementary Appendix 1. Exclusion criteria**

Patients who met any of the following criteria were not eligible for the study:

1. A woman who was breastfeeding, was pregnant or may be pregnant, or was unable to give consent to contraception by medically appropriate means for up to 7 days after administration of the study drug. Medically appropriate contraception referred to the use of two or more of the following: no sexual intercourse, surgical sterilization by vasectomy, etc. or intrauterine devices, oral contraceptives, or condoms.
2. Patients with serious hepatic disorder (aspartate transaminase or alanine transaminase at the time of eligibility assessment more than three times the upper limit of the reference level of the trial site and total bilirubin more than two times the upper limit of the reference level of the trial site) or renal disorder (estimated glomerular filtration rate at eligibility assessment: 30 mL/min/1.73 m^2^ or less).
3. Individuals with hypersensitivity to ivermectin.
4. Individuals with a history of serious drug allergy such as Stevens-Johnson syndrome or toxic epidermal necrolysis.
5. Individuals who had received prohibited concomitant medications within the past month (within the past 6 months for biologics) or who required the use of prohibited concomitant medications during the study period.
6. Individuals scheduled for SARS-CoV-2 vaccination between the date of informed consent and the completion of follow-up (as vaccination may impact disease severity).
7. Participants who were part of other clinical trials or who had participated in other clinical trials within 30 days before obtaining informed consent. In addition, the investigator or sub‑investigator judged whether a patient was inappropriate for this clinical trial.

**Supplementary Appendix 2. Seven-point ordinal scale for assessing change in disease status**

| ① | No pneumonitis and no limitation in activities of daily living (ECOG-PS 0) |
| --- | --- |
| ② | No pneumonitis, limited activities of daily living (ECOG-PS [1] or above) |
| ③ | With pneumonia, no oxygen support required |
| ④ | With pneumonia, any oxygen support required |
| ⑤ | With noninvasive assisted ventilation or high-flow oxygen support |
| ⑥ | With mechanical ventilation management (including ECMO use) |
| ⑦ | Death |

Pneumonia was defined as the appearance of shadows on a plain chest radiograph or CT scan of the chest along with no obvious condition other than pneumonia, such as heart failure, as the underlying cause.

Criteria for limited activities of daily living: 0 is defined as no limitation in activities of daily living and 1 or more is defined as limited activities of daily living according to the criteria presented in the below table:

**ECOG-PS** [1]

| **Score** | **Definitions** |
| --- | --- |
| 0 | Fully active, able to carry on all pre-disease performance without restriction |
| 1 | Restricted in physically strenuous activity but ambulatory and able to carry out work of a light or sedentary nature, e.g., light housework, office work |
| 2 | Ambulatory and capable of all selfcare but unable to carry out any work activities; up and about more than 50% of waking hours |
| 3 | Capable of only limited selfcare; confined to bed or chair more than 50% of waking hours |
| 4 | Completely disabled; cannot carry on any selfcare; totally confined to bed or chair |
| 5 | Dead |

CT, computed tomography; ECMO, extracorporeal membrane oxygenation; ECOG-PS, Eastern Cooperative Oncology Group performance status.

**Supplementary Appendix 3.** **Data collected using an electronic device and the number of ivermectin tablets administered by patient body weight**

Patients with their own cellphones installed an application specific for this study and those without cellphones were provided an electric device with the application preinstalled. Each patient was provided a pulse oximeter and a thermometer. Patients were instructed to measure their vital signs and share this information along with their responses to the patient-reported outcome questionnaires through the application.

Number of ivermectin tablets administered by patient body weight

| Weight (kg) | Number of ivermectin tablets administered |
| --- | --- |
| 40–50 | 3 |
| 51–65 | 4 |
| 66–80 | 5 |
| 81–95 | 6 |
| 96–110 | 7 |
| 111–125 | 8 |
| 126–140 | 9 |
| 141–155 | 10 |

**Reference**

1. Oken MM, Creech RH, Tormey DC, Horton J, Davis TE, McFadden ET, et al. Toxicity and response criteria of the Eastern Cooperative Oncology Group. *Am J Clin Oncol*. (1982) 5:649–55.
